# Reverse Engineering the Gap Gene Network of Drosophila melanogaster

**DOI:** 10.1371/journal.pcbi.0020051

**Published:** 2006-05-19

**Authors:** Theodore J Perkins, Johannes Jaeger, John Reinitz, Leon Glass

**Affiliations:** 1 McGill Centre for Bioinformatics, McGill University, Montreal, Quebec, Canada; 2 Department of Applied Mathematics and Statistics, Stony Brook University, Stony Brook, New York, United States of America; 3 Center for Developmental Genetics, Stony Brook University, Stony Brook, New York, United States of America; 4 Centre for Nonlinear Dynamics, Department of Physiology, McGill University, Montreal, Quebec, Canada; Millennium Pharmaceuticals, United States of America

## Abstract

A fundamental problem in functional genomics is to determine the structure and dynamics of genetic networks based on expression data. We describe a new strategy for solving this problem and apply it to recently published data on early Drosophila melanogaster development. Our method is orders of magnitude faster than current fitting methods and allows us to fit different types of rules for expressing regulatory relationships. Specifically, we use our approach to fit models using a smooth nonlinear formalism for modeling gene regulation (gene circuits) as well as models using logical rules based on activation and repression thresholds for transcription factors. Our technique also allows us to infer regulatory relationships de novo or to test network structures suggested by the literature. We fit a series of models to test several outstanding questions about gap gene regulation, including regulation of and by *hunchback* and the role of autoactivation. Based on our modeling results and validation against the experimental literature, we propose a revised network structure for the gap gene system. Interestingly, some relationships in standard textbook models of gap gene regulation appear to be unnecessary for or even inconsistent with the details of gap gene expression during wild-type development.

## Synopsis

Modeling dynamical systems involves determining which elements of the system interact with which, and what is the nature of the interaction. In the context of modeling gene expression dynamics, this question equates to determining regulatory relationships between genes. Perkins and colleagues present a new computational method for fitting differential equation models of time series data, and apply it to expression data from the well-known segmentation network of *Drosophila melanogaster.* The method is orders of magnitude faster than other approaches that produce fits of comparable quality, such as Simulated Annealing. The authors show that it is possible to detect interactions de novo as well as to test existing regulatory hypotheses, and they propose a revised network structure for the gap gene system, based on their modeling efforts and on other experimental literature.

## Introduction

**Figure 1:**
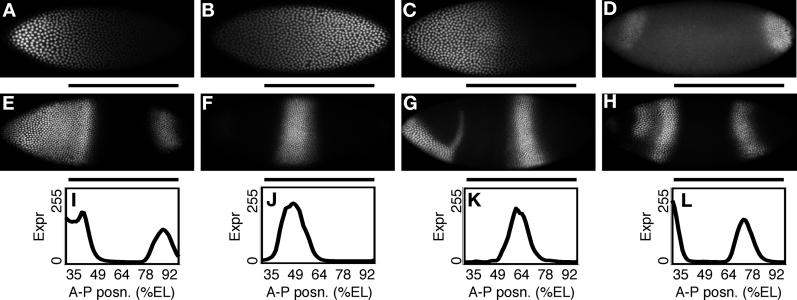
Maternal and Gap Gene Expression (A–C) *Drosophila* embryos at early blastoderm stage (cleavage cycle 13) fluorescently stained for Bcd (A), Cad (B), and Hb (C) protein. (D–H) *Drosophila* embryos at late blastoderm stage (late cleavage cycle 14A) fluorescently stained for Tll (D), Hb (E), Kr (F), Kni (G), and Gt (H) protein. Anterior is to the left, dorsal is up. Black bars indicate the A–P extent we model. (I–L) Mean relative gap protein concentration as a function of A–P position (measured in percent embryo length) for Hb (I), Kr (J), Kni (K), and Gt(L). Expression levels are from images and are unitless, ranging from 0 to 255. Images and expression profiles are from the FlyEx database [18]. Embryo IDs: bd3 (A,B), hz30 (C), tb6 (D), kf9 (E), kd17 (F), fq1 (G), nk5 (H).

The segmented body pattern of Drosophila melanogaster is established by the expression of segmentation genes during the blastoderm stage of development (reviewed in [1,2]). The polarity of the embryo along its anterior–posterior (A–P) axis is established by maternal gradients of the Bicoid (Bcd), Hunchback (Hb), and Caudal (Cad) transcription factors (Figure 1A–1C). The trunk gap genes, *hunchback (hb), Krüppel (Kr)*, *knirps (kni),* and *giant (gt),* are among the earliest targets of these maternal gradients. They show broad, overlapping expression domains (Figure 1E–1H). Gap genes together with maternal factors then regulate the expression of downstream targets, such as pair-rule and segment-polarity genes, which establish the segmental periodicity of the *Drosophila* body plan.

The gap gene network has been studied extensively by means such as the analysis of transcription factor binding sites on the DNA and the measurement of gene expression under wild-type and mutant conditions. These studies are the basis of qualitative regulatory models (e.g., [3,4]), which assert activating or repressing regulatory relationships between genes. These models provide a useful summary of the interactions within the network. However, ambiguities in the primary data can lead to different qualitative models. For example, if deleting a gene *A* results in decreased expression of a gene *B,* is *A* necessarily an activator of *B*? Or, could *A* be a repressor of some other gene *C,* which in turn represses *B*? This particular type of confusion has resulted in conflicting models of at least two relationships in the gap gene system, regarding the effect of Hb on *Kr* and the effect of Kr on *kni*. Qualitative models are also limited in that they usually do not specify precisely how conflicting regulatory signals (e.g., activation and repression) are resolved, leaving one without means for predicting the results of genetic or pharmacological interventions, for example. Further, qualitative models typically provide little or no explanation of the timing or dynamics of expression, which are key questions in processes such as development.

The increasing availability of quantitative gene expression data raises the possibility of detailed mathematical modeling of the regulatory relationships between genes. Indeed, recent work has shown that model parameters and even the existence of regulatory relationships can be automatically inferred from expression time series data [5–9]. Quantitative models can sometimes resolve ambiguities in qualitative models, offer much more specific predictions, and allow in-depth analysis of temporal changes in expression and the dynamical roles of regulatory relationships. For example, recent work on the gap gene system [6] has highlighted previously unappreciated effects of known regulatory relationships in shifting the expression domains of *Kr, kni,* and *gt*.

Unfortunately, fitting quantitative dynamical models can be computationally challenging. Only two techniques [5,10–12] have so far proven capable of fitting satisfactory models of the spatio–temporal segmentation gene expression data that we study, and both involve very long running times. Fitting all the models for the recent Jaeger et al. study [6,7], for example, which was done by parallel simulated annealing [5,10,11], required an estimated 20,000 CPU hours, or approximately two CPU years. Because of these intensive computing requirements, alternative regulatory explanations were not explicitly explored, and it has not been established whether previously proposed regulatory models (e.g., [3]) are equally capable of capturing the data. Long fitting times also make it difficult to explore alternative mathematical forms for the model. Gene regulation is an enormously complex process, and it is unclear in general how much of the biochemistry can be simplified away while retaining the key properties of the system. At a coarse level, the behavior of some genetic networks appears adequately captured by Boolean (on/off) models of gene expression [13–15]. However, detailed analysis of some genes has revealed more complex behavior which may well be functionally relevant [16,17]. Thus, it is advantageous to be able to easily experiment with different forms of models.

We use a novel strategy for fitting network models to spatio–temporal gene expression data that is compatible with a variety of modeling formalisms and, by combining function approximation techniques with simulation-based optimization, is vastly faster than previous methods [5,10–12]. The increased efficiency of the algorithm enables the fitting of many more models, allowing us to explore alternative modeling formalisms and assumptions about regulatory relationships between genes. We applied this strategy to fit four models to gap gene expression data [18]. Together, these models demonstrate different modeling formalisms and address alternative regulatory explanations for gap gene expression. Specifically, we show that both a smooth nonlinear representation of gene regulation (gene circuits [5–7]) and a discontinuous logical formalism based on activation and repression thresholds are capable of reproducing the observed gap gene expression dynamics, and that such models can efficiently be fit to the data. We show that it is possible to infer the activating and repressing relationships between genes directly from the data, as well as to test specific network structures, namely the model of Rivera-Pomar and Jäckle [3]. We find that the Rivera-Pomar and Jäckle model does not correctly capture all the major features of gap gene expression and requires additional regulatory links. Further, our optimizations consistently eliminate certain links in the Rivera-Pomar and Jäckle model, suggesting that those links are incorrect. (In Protocol S1, we also report on fits using the more recent but similar Sanchez and Thieffry network structure [4], and find similar conclusions.) We summarize our findings in a Combined network model that includes a core of regulatory relationships found in all of our models in addition to regulatory relationships that are supported by some models and that are consistent with other experimental work.

## Results/Discussion

### Modeling Gap Gene Expression Dynamics

Gap gene expression is established in the trunk region of the embryo, indicated by the black bars in Figure 1, during cleavage cycles 13 and 14A (before cellularization), a span of approximately 70 min. We model the wild-type expression of the four trunk gap genes, *hb, Kr, kni,* and *gt* (Figure 1C and 1E–1H). We do not model the expression of *bcd, cad,* or *tailless (tll),* a terminal gap gene (Figure 1A, 1B, and 1D). However, we allow the observed expression values for these genes to act as regulatory input to our models of the trunk gap genes. Expression of these genes is largely invariant along the dorsal–ventral axis of the embryo. Thus, we model expression as a function of time and of position along the A–P axis (Figure 1I–1L). At the start of cleavage cycle 13, there is some *hb* expression in the anterior, due to maternally deposited *hb* mRNA. There is no unambiguously detectable expression for the other three gap genes. By the end of cleavage cycle 14A, there are six main gap expression peaks, two each for *hb* and *gt* and one each for *Kr* and *kni*.

We represent gap gene dynamics using a reaction–diffusion partial differential equation [12]:


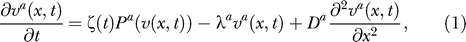


where *v^a^*(*x*,*t*) denotes the relative concentration of gap protein a (unitless, ranging from 0 to 255) at space point *x* (from 35% to 92% of embryo length) and time *t* (0 min to 68 min after the start of cleavage cycle 13). The three terms on the right-hand side account for production, decay, and diffusion of protein, respectively. *λ^a^* and *D^a^* are decay and diffusion rates. The model combines the processes of transcription and translation into a single production process. *P^a^* specifies the production rate of protein *a,* as a function of the vector of concentrations of all proteins (including Bcd, Cad, and Tll) at the same point in space and time, *v*(*x*,*t*). The factor *ζ*(*t*) accounts for changes in transcribing gene density, due to the shutdown of transcription during mitosis [19] and due to the doubling of nuclei (see Materials and Methods for details).

We fit a total of four models, resulting from two different choices for the form of the production rate functions, *P^a^,* and two different sets of constraints on the regulatory relationships between genes. The first model uses a gene circuit formalism identical to the one used by Jaeger et al. [6,7] for the production rate functions.


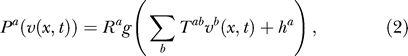


where *R^a^* is the maximum production rate, *b* ranges over the seven genes {*bcd, cad, hb, Kr, gt, kni, tll*}, and 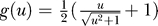 . The regulatory weights, *T^ab^,* encode the effect protein *b* has on the production rate of protein *a*. If *T^ab^* > 0, then we interpret gene *b* as being an activator of gene *a*. If *T^ab^* < 0, then we say it is a repressor. We place no restrictions on the signs of the regulatory weights, so that the optimization determines the qualitative regulatory relationships between the genes. We call the resulting model Unc-GC, for unconstrained gene circuit. Following Jaeger et al. [6,7], we fix the bias, or offset, terms *h^a^* = −3.5 for all *a*.

While our first model tests the feasibility of inferring regulatory relationships de novo, our second model tests the ability of an established regulatory model to reproduce the data. In our second gene circuit model, regulatory relationships are limited to those in the model of Rivera-Pomar and Jäckle [3], with one exception. Rivera-Pomar and Jäckle did not consider the posterior *hb* domain, and their regulatory relationships cannot reproduce this domain (unpublished data). So, we added Tll activation of *hb,* which was sufficient to activate a posterior *hb* domain. We call this set of activation and repression relationships the RPJ network structure. The weights that represent these relationships are constrained to be positive or negative for relationships that are respectively activating or repressing. An additional complication in the RPJ network structure is that *Kr* is activated by low levels of Hb but repressed by high levels of Hb. We model this by allowing two weights for describing the effect of Hb on *Kr*—a positive weight that is multiplied by *v^hb^*(*x*,*t*) and a negative weight that is multiplied by (*v^hb^*(*x*,*t*))^2^/255. We call the resulting model RPJ-GC.

Previous analyses suggest that the general regulatory principle of the gap gene system is that genes are activated in broad regions by maternal gradients, but that repression from other gap genes can locally overwhelm general activation [3,7]. Our final two models rely on logical rules for the production rate functions that implement this general plan. Specifically, we assume that gap protein *a* is produced at rate *R^a^* if at least one of its activators exceeds an activation threshold and none of its repressors exceed their repression thresholds. For example, the rule


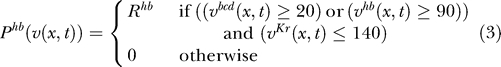


states that Bcd is an activator of *hb, hb* is autoactivating, and Kr is a repressor. If there is enough Bcd or Hb and not too much Kr, as determined by the activation and repression thresholds, then there is production of Hb at the rate *R^hb^*.

For our logical models, fitting the production rate functions means finding values for the *R^a^* and for the activation and repression thresholds. We do not attempt to optimize which relationships are activating and which are repressing, although the optimization can change a threshold so that the activating or repressing effect is effectively eliminated. Our Unc-Logic model employs the regulatory structure discovered by our unconstrained gene circuit fit, except that we remove Gt activation of *hb* and Kni activation of *gt* (see Materials and Methods for justification). Our RPJ-Logic model uses the RPJ regulatory relationships.

### Model Fitting

**Figure 2:**
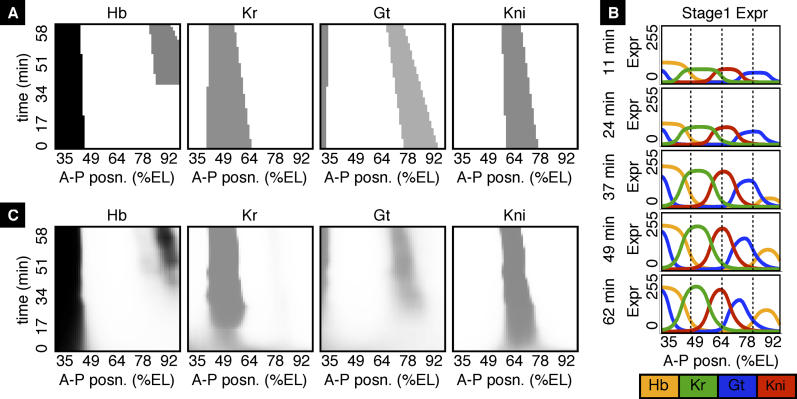
Outline of the Optimization Approach In Stage 1, protein production associated to each domain is assumed to fall within a quadrilateral-shaped region of space–time (A) (darkness indicates rate of production), whose boundaries are optimized so that simulated expression (B) matches observed data (Figure 3B). In Stage 2, regulatory parameters are estimated by trying to fit the quadrilateral production regions (A) based on the observed levels of transcription factors present at each space–time point (C). In Stage 3, local search, starting from the parameter values estimated in Stage 2, is used to optimize a fully coupled partial differential equation model of gene expression, so that simulated expression (Figure 3C–3F) matches observed expression (Figure 3B).

We fit all four models using the same general strategy outlined in Figure 2 and described in greater detail in Materials and Methods and Protocol S2. In short, the strategy has three stages. Stage 1 produces an initial estimate of the decay and diffusion parameters for each gene and the spatial and temporal extents of protein production associated with each expression domain, without any regulatory explanation (Figure 2A and 2B). Stage 2 produces an initial estimate of the regulatory parameters by attempting to fit the estimated protein production rate at each space–time point (from Stage 1) in terms of the observed protein levels present (Figure 2C). In Stage 3, all model parameters are optimized using a local search procedure, starting from the initial regulatory, decay, and diffusion parameter estimates produced in the first two stages. The optimization approach we use in the third stage is much simpler than the approach used by Jaeger et al. [5–7,10,11]. However, if the initial parameter estimates are sufficiently good, then it can produce results of comparable quality and can do so relatively quickly.

**Figure 3:**
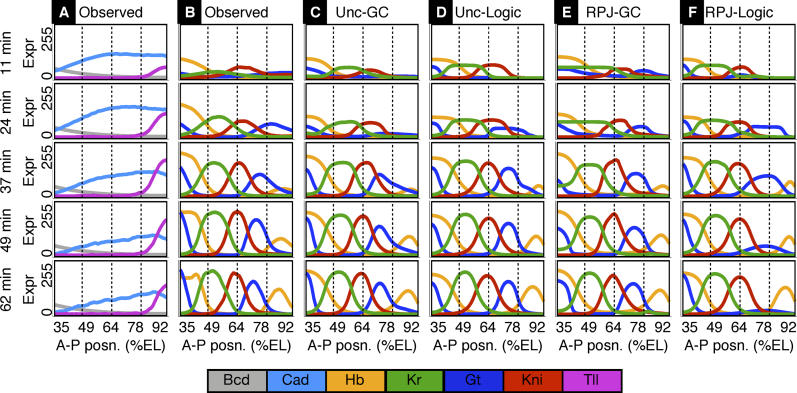
Observed and Simulated Expression at Five Time Points (A) Observed expression of *bcd, cad,* and *tll,* which are not modeled, but which are allowed to act as exogenous inputs to the trunk gap gene models. (B) Observed expression of the trunk gap genes. (C–F) Simulated expression produced by the models Unc-GC, Unc-Logic, RPJ-GC, and RPJ-Logic, respectively. The horizontal axis in each plot is A–P position, ranging from 35% to 92% of embryo length. The vertical axis represents relative protein concentration corresponding to fluorescence intensity from quantitative gene expression data [18] (units arbitrary).

### Optimization Results

The observed expression data is shown in Figure 3A and 3B. Simulated gap gene expression from our best-fitting models of each type is shown in Figure 3C–3F. The root mean squared errors (RMS error; see Materials and Methods) of these models are, in order of best to worst, 12.29 (Unc-GC), 14.83 (Unc-Logic), 15.88 (RPJ-GC), and 21.91 (RPJ-Logic). The Unc-GC and Unc-Logic models reproduce all six expression domains in approximately the right locations and with approximately the right timing (Figure 3C and 3D). The RPJ-Logic model suffers a major failure. It begins to form, but does not sustain, the posterior *gt* domain (Figure 3F). This accounts for much of the difference in RMS error between that model and the other four. The RPJ-GC model also contains a significant error, although it eventually reproduces all six domains. At the early time points, Kr production extends through the whole front of the embryo (Figure 3E, *t* = 11,24 min). Only later does repression from Hb eliminate production in the anterior, so that *Kr* is expressed in the correct region. The RPJ-GC model also creates a small erroneous *Kr* domain in the posterior half of the trunk (Figure 3E, *t* = 62 min). This is discussed further below.

#### Summary of regulatory mechanisms in the models.

**Figure 4:**
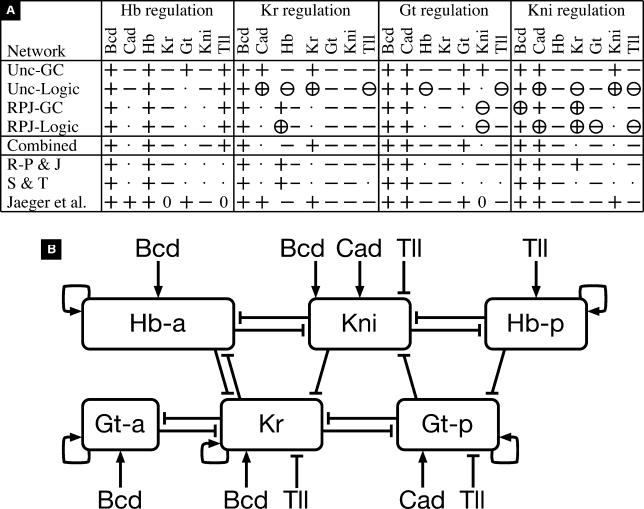
Regulatory Relationships in Our Models and Previously Published Models (A) Qualitative regulatory relationships in our four models after optimization (Unc-Gc, Unc-Logic, RPJ-GC, and RPJ-Logic), a Combined model (see text for details), and the relationships posed by Rivera-Pomar and Jäckle (R-P & J) [3], Sanchez and Thieffry (S & T) [4], and Jaeger et al. [6,7]. “+” represents activation, “−” represents repression, “+−” represents activation at low levels of the regulator and repression at high levels, “·” represents no regulatory relationship. For Jaeger et al., “0” represents a regulatory relationship that was eliminated by optimization. For our models, we use “⊕” and “⊝” to denote activating and repressing relationships, respectively, that were eliminated by optimization. For the gene circuit models, a regulatory relationship was considered eliminated if the corresponding weight was 0.0001 or less in magnitude. For the logical models, an activation or repression term was considered to be eliminated if removing it resulted in no change in simulated expression. (B) Diagram of the Combined network model. Boxes represent trunk gap gene domains, with endings “-a” or “-p” denoting the anterior and posterior domains, respectively, for *hb* and *gt*. Arrows (→) indicate activation while T-connectors (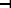) represent repression.

**Figure 5:**
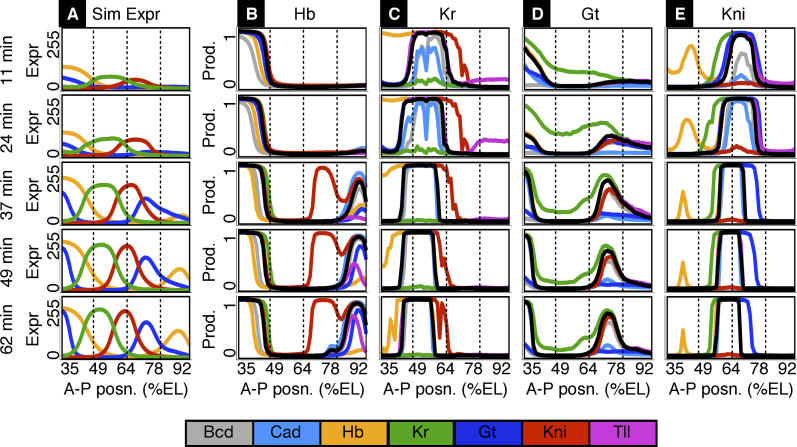
Simulated Gap Gene Expression, Production, and Regulatory Effects in the Unc-GC Model (A) Simulated expression at five of the 10 times for which we have observed data. (B–E) Production rates of Hb, Kr, Gt, and Kni, respectively, as a fraction of maximum (black curve) along with the production rate that would result when individual regulatory inputs are removed (colored curves). For example, in the plot for Hb at time *t* = 62 min, the yellow curve below the black curve shows what the production rate of Hb would be if, at that moment, Hb autoactivation were removed from the model, and the red curve shows what the production rate would be if repression by Kni were removed.

**Figure 6:**
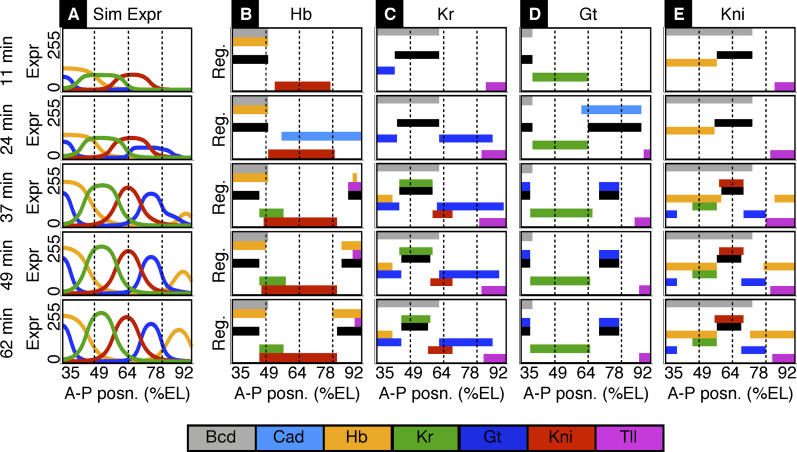
Simulated Gap Gene Expression, Production, and Regulatory Effects in the Unc-Logic Model In each plot in columns B–E, the black bar indicates the spatial extent of production. Colored bars above the black bar represent regions in which the corresponding activatory input is above threshold (at least one activator must be above threshold for production to occur). Colored bars below the black bar represent regions in which the corresponding repressive input is above threshold (production only occurs if no repressors are above threshold).

**Figure 7:**
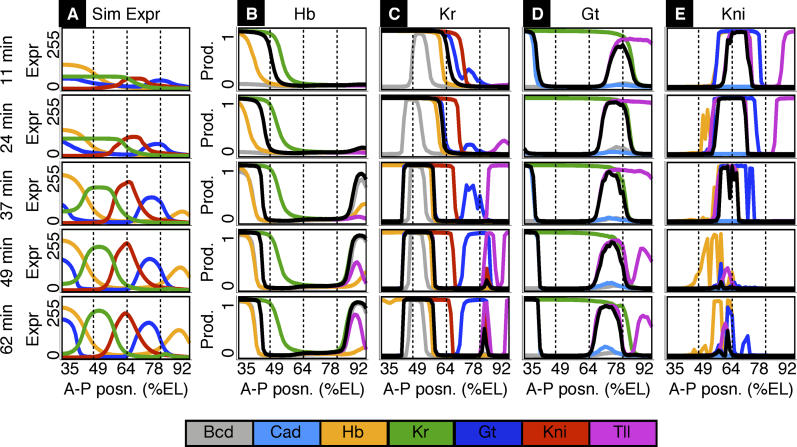
Simulated Gap Gene Expression, Production, and Regulatory Effects in the RPJ-GC Model

**Figure 8:**
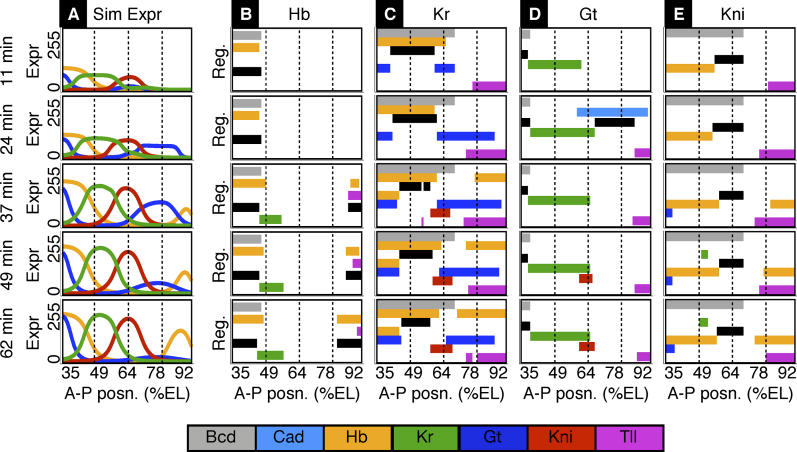
Simulated Gap Gene Expression, Production, and Regulatory Effects in the RPJ-Logic Model

The exact parameters for the best-fitting models of each type can be found in Protocol S3. The qualitative relationships they represent, as well as the relationships in some previously published models, are summarized in Figure 4A. Figures 5–8 provide detailed snapshots of the regulatory action between genes at different times. The models agree on many of the qualitative relationships between genes and never contradict each other, in the sense of one model claiming a relationship is activating while another claims it is repressing.

Figure 4 also includes a Combined network model and diagram, which represents our best estimate of the regulatory relationships in the real system. The Combined model includes a regulatory relationship if it was deemed significant in all of our models or if it was significant in it at least some of our models, performing a correct function, and is supported by other experimental evidence, such as binding site analysis or mutant or overexpression studies. Weights in a gene circuit model are considered insignificant if they are smaller than 0.0001 in magnitude. Terms in a logical model are considered insignificant if their removal results in no change in simulated expression. We adopted these stringent conditions on insignificance because we preferred to make decisions about weak/borderline interactions based on other experimental evidence rather than, for example, choosing some arbitrary threshold. (See the final paragraph of the Results and Discussion section for more information.) The regulatory structure of the Combined model is itself sufficient to reproduce all six gap gene domains using either the gene circuit or logical formalisms for production rate functions (Protocol S4). We first cite support for our Combined model, and then consider the results of the individual models in light of several outstanding questions about gap gene regulation.

The maternal proteins Bcd and Cad are largely responsible for activating the trunk gap genes, with Bcd being more important for the anterior domains and Cad more important for the posterior domains. Bcd is a primary activator of the anterior *hb* domain [20,21], the anterior *gt* domain [22], and the *Kr* domain [23,24]. Cad activates posterior *gt* [25]. The *kni* domain is present in *bcd* mutants and in *cad* mutants, but not in *bcd*;*cad* double mutants. This suggests redundant activation by the two maternal factors. Such redundant activation of *kni* is present in our Unc-GC model. For the other models, the optimization selected one or the other as activators, but not both. Tll is crucial for activating the posterior *hb* domain [26–28], while it represses *Kr, kni,* and *gt,* preventing their expression in the extreme posterior [22,29–34]. All the regulatory relationships between the gap genes in our Combined model are repressive. The complementary gap gene pairs, *hb*–*kni* and *Kr*–*gt* are strongly mutually repressive [22,32,35–38], as was found in nearly all of our models. (Repression of *hb* by Kni is not part of the RPJ regulatory relationships, but our Unc-GC and Unc-Logic models included the link.) Our models also suggest that mutual repression between *hb* and *Kr* helps to set the boundary between those two domains [38–41]. A chain of repressive relationships, *hb⊣gt⊣kni⊣Kr* [6,7], causes the shifts in the *Kr, kni,* and posterior *gt* domains. Autoactivation by *hb* is well-established [42], and there is also some evidence for autoactivation by *Kr* [43] and *gt* [22].

#### Does Hb have a dual regulatory effect on *Kr*?

There is a long-running debate about whether or not low levels of Hb activate *Kr*. In *hb* mutants, the *Kr* domain expands anteriorly, suggesting that Hb represses *Kr* [39]. However, *Kr* expression in these mutants is lower than in wild-type [31] and expands posteriorly in embryos overexpressing Hb [36]. Further, in embryos lacking Bcd and Hb, the *Kr* domain is absent, but can be restored in a dosage-dependent manner by reintroducing Hb [44,45]. These observations suggest that Hb activates *Kr*. It has been suggested, therefore, that low levels of Hb activate *Kr* while high levels repress it [36,44,45]. An alternative explanation, however, is that the apparently activating effects of Hb are indirect, via Hb's repression of *kni* and Kni's repression of *Kr* [7]. Optimization of the Unc-GC model, which could have resulted in activation or repression of *Kr* by Hb, but not both, resulted in repression (Figure 4A). The RPJ models allow for a dual effect, but activation by Hb was eliminated during optimization of the RPJ-Logic model. The RPJ-GC model retained functional activation and repression of *Kr* by Hb. However, *Kr* expression in this model is defective. *Kr* is not properly repressed in the anterior (Figure 3E, *t* = 11,24 min). Further, *Kr* is ectopically expressed in a small domain in the posterior of the embryo (Figure 3E, *t* = 62 min and Figure 7C, *t* = 62 min). Thus, our models provide no support for activation of *Kr* by Hb. The only support we find, which is crucial in all models except Unc-Logic and also consistent with the mutant and overexpression studies cited above, is for repression of *Kr* by Hb.

#### What represses *hb* between the anterior and posterior domains?

Another point of disagreement in the literature is what prevents the expression of *hb* between its two domains. In the model of Rivera-Pomar and Jäckle [3], repression by Kr is the explanation. Our RPJ models confirm that this mechanism is sufficient. Specifically, in these models Kr repression prevents *hb* expression just to the posterior of the anterior *hb* domain (Figures 7B and 8B, *t* = 37,49,62 min). Between the *Kr* and posterior *hb* domains, there is no explicit repression of *hb*. Rather, Hb is not produced simply because of a lack of activating factors. In contrast, the models of Jaeger et al. [6,7] detected no effect of Kr and attributed repression solely to Kni. Our Unc-GC and Unc-Logic models found repression by Kni, but in addition to repression by Kr, not instead of it (Figures 5B and 6B). Kr is more responsible for repression near the anterior *hb* domain and Kni is more responsible for repression near the posterior *hb* domain. This is consistent with observations of expression in mutant embryos. Embryos mutant for *Kr* show slight expansion of the anterior *hb* domain [38], while *kni* embryos show expansion of the posterior *hb* domain [39]. In *Kr*;*kni* double mutants, *hb* is completely derepressed between its two usual domains [38]. This suggests, as seen in our Unc-GC and Unc-Logic models, that Kr and Kni are both repressors of *hb*, that their activity is redundant in the center of the trunk, and that Kr and Kni are the dominant repressors for setting the boundaries of the anterior and posterior domains, respectively. This interpretation was also favored by Jaeger et al. [6,7], on the basis of the mutant data, even though their models did not find repression by Kr.

#### The posterior *hb* domain.

In all of our models, the posterior *hb* domain is activated by Tll and sustained by Tll and *hb* autoactivation (Figures 5–8B, *t* = 37,49,62 min). Rivera-Pomar and Jäckle [3] did not consider the posterior *hb* domain, and did not include activation by Tll in their model. We added that link to the RPJ network structure because otherwise it was not possible to capture the posterior *hb* domain (unpublished data). The model of Jaeger et al. [6,7] captured the domain without Tll activation by substituting activation from *cad*. However, there is no confirming evidence for such an interaction. The absence of posterior *hb* in *tll* mutants [26–28] and the inability of our models to explain posterior *hb* by other means, leads us to believe the straightforward hypothesis that Tll activates posterior *hb*. Posterior *hb* is unique in that the domain begins to form later than the other five domains we model (Figure 3B, *t* = 37 min). In our RPJ models, this happens simply because high levels of Tll are needed to activate *hb*—levels that are reached only at about *t* = 30 min (Figure 3A, *t* = 24,37 min). The Unc-GC and Unc-Logic models also employ repression by Cad to slightly delay Hb production in the posterior (Figures 5B, *t* = 24 min, and 6B, *t* = 24 min). However, there is no confirming evidence for such repression, and we omit it from our Combined model.

#### Shifting of the *Kr, kni,* and posterior *gt* domains.

Domain shifting was first observed by Jaeger et al. [6,7] and attributed to a chain of repressive regulatory relationships, *hb⊣gt⊣kni⊣Kr*. Our models largely support the importance of this regulatory chain, particularly the final two links. Repression of *Kr* by Kni was significant in all of our models (Figure 4A). Repression of *kni* by Gt was present in all models except RPJ-Logic, where it would be of little impact anyway, as RPJ-Logic has a defective posterior *gt* domain. Consistent with these findings, Kni binds to the regulatory region of *Kr* [29], and the *Kr* domain expands towards the posterior in *kni* mutants [39,40]. Similarly, the *kni* domain expands posteriorly in *gt* mutants [22], while embryos overexpressing *gt* show reduced *kni* expression [35].

Repression of *gt* by Hb is not as well supported by our models. The Unc-GC model included the link, though the regulatory weight was the smallest of all those in the model (Protocol S3). The link was eliminated from Unc-Logic and, of course, not present in the RPJ network structure. Instead, the models utilized decreasing activation by Cad (Unc-GC, Unc-Logic) and repression by Tll (Unc-GC, RPJ-GC) to shift the posterior *gt* domain (Figures 5–7D). Even with these links, however, shifting of the domain is not well-captured (Figure 3C, 3D, and 3F). RPJ-GC appears to capture the posterior *gt* shift best (Figure 3E). However, it relies on its small ectopic *Kr* domain to repress *gt,* a completely incorrect mechanism (Figure 7D, *t* = 62 min). Interestingly, a gene circuit fit using the network structure of Sanchez and Thieffry [4] (Protocol S1), captured the shift of posterior *gt* better than any of our other models, and it did so using repression of *gt* by Hb, providing additional modeling support for the relationship. There also is strong mutant evidence in favor of the relationship. In *hb* mutants, the posterior *gt* domain does not retract from the posterior pole [22,32,46]. Further, Gt is absent in embryos that have ubiquitous Hb, such as maternal *oskar* or *nanos* mutants [22,32] or embryos expressing Hb ubiquitously under a heat-shock promoter [33]. Thus, we find sufficient evidence to include a repressive link from *hb* to *gt* in our Combined model.

Activating or repressing links that oppose the direction of the repressive chain were eliminated by optimization of the Unc-Logic, RPJ-GC, and RPJ-Logic models (Figure 4A). In agreement with this result, the boundaries of the *kni* and posterior *gt* domains are correctly positioned in *Kr* and *kni* mutants, respectively [22,31,37,46]. Thus, the simplest picture supported by our models and consistent with the mutant studies is that there is no regulation from *Kr*, *kni,* or posterior *gt* to any of their immediate posterior neighbors, and that the repressive chain highlighted by Jaeger et al. [6,7] is indeed responsible for domain shifting.

#### Do gap genes autoregulate?

All four of our models include autoactivation by *hb*. This is supported by the observation that late anterior *hb* expression is absent in embryos lacking maternal and early zygotic Hb [47]. Our models suggest *hb* autoactivation also plays a crucial role in sustaining the posterior domain, once it has been initiated by Tll (Figures 5–8B, *t* = 37–62), a role not previously emphasized. Autoactivation for the other genes was found by our Unc-GC model, but is not part of the RPJ network structure (Figure 4A). We included autoactivation only for *Kr* and *gt* in our Combined model, on the basis of a weakened and narrowed *Kr* domain in embryos producing defective *Kr* protein [43] and a delay in *gt* expression in embryos producing defective *gt* protein [22]. Interestingly, the gene circuit models of Jaeger et al. [6,7] also found autoactivation for all four gap genes, but they considered autoactivation by *gt* to be the weakest and least certain. In contrast, our Unc-Logic model retained *gt* autoactivation while eliminating autoactivation for *Kr* and *kni* (Figure 4A). The RPJ-Logic model was unable to reproduce the posterior *gt* domain. However, we found that by adding *gt* autoactivation to the model, it was able to create and sustain posterior *gt* correctly, bringing the error of the model down to 15.34 (unpublished data). This suggests that, after *hb, gt* is the most likely candidate for autoactivation. However, even this is not strictly necessary. The RPJ-GC model is able to reproduce and sustain the posterior *gt* domain without autoactivation by relying on cooperative activation from Bcd and Cad (Figure 7D).

### Conclusions

#### Comparison of regulatory architectures*.*

The regulatory relationships proposed by Rivera-Pomar and Jäckle [3] are not fully consistent with the data and require amending. Repression of *gt* by Kni, which contradicts the mechanism of domain shifts described by Jaeger et al. [6,7], was eliminated by the optimization in both of our models based on the RPJ regulators. We also never observed activation of *kni* by Kr. We found no support for a dual regulatory effect of Hb on *Kr*. Activation of *Kr* at low levels of Hb was eliminated in the RPJ-Logic model. It was retained in the RPJ-GC model, but resulted in serious patterning defects. Inclusion of Tll as an activator of *hb* was sufficient to produce the posterior *hb* domain. Based on our fits and the primary experimental literature, there are likely other regulatory links missing from the model of Rivera-Pomar and Jäckle, though they are not strictly required to reproduce the wild-type gap gene patterns. Foremost is repression of *hb* by Kni, which appears important for eliminating *hb* expression anterior of the posterior domain. Fits based on the Sanchez and Thieffry regulatory relationships [4] (Protocol S1) also support these conclusions.

In contrast, the regulatory relationships in our Combined model and both the Unc-GC and Unc-Logic models are able to capture the wild-type gap patterns without gross defects. The relationships in the Unc-GC model are very similar to those obtained by Jaeger et al. [6,7]. For example, the regulation of *Kr* and *kni* is qualitatively equivalent in both models, and there is a single minor difference in the regulation of *gt* (Figure 4A). Our optimizations correctly identified activation of *hb* by Tll, which was missed by Jaeger et al. [6,7], though our models did less well at capturing shifting of the posterior *gt* domain. These regulatory relationships are also similar to those found by Gursky et al. [12], though that study was based on gap gene expression data with much lower accuracy and temporal resolution than the data used here [5,18]. These similarities show that differences in the mathematical formulations of these models—as ordinary versus partial differential equations, how diffusion and nuclei doubling are modeled, and choice of boundary conditions and other simulation parameters—are not important for the reproduction of the gap gene patterns nor for the inference of regulatory relationships from the data.

#### Comparison of gene circuit and logical formalisms*.*

Both of our gene circuit models fit the data better than the corresponding logical models. Although both types of models grossly simplify the complexity of gene regulation, this suggests that the gene circuit formalism is a better description of gap gene regulation than the logical formalism. However, the Unc-Logic model shows that the logical formalism can correctly capture the main features of gap gene expression. Of greater concern is that the strict on/off nature of the logical rules renders many regulatory inputs completely redundant, effectively eliminating them from the regulatory structure (Figures 4A, 6, and 8). In sparsely connected gene networks, this may be a useful bias. In a densely connected network like the gap gene system, it results in the elimination of many correct regulatory links. Another serious drawback of the logical models is that we could not find a satisfactory method for inferring the regulatory structure as part of the optimization process. We were forced to set the activators and repressors in the Unc-Logic model from the Unc-GC findings, and were able only to optimize the strengths of the regulatory relationships, represented by the activation and repression thresholds. Resolving this problem is an important avenue for further work.

#### Speed and accuracy of the fitting method*.*

The RMS errors in simulated expression for the Jaeger et al. [6,7] model and our Unc-GC model are comparable, so both optimization algorithms are equally successful in fitting the models to the data (Protocol S5). An important advantage of our technique, however, is speed. Each run of the algorithm used by Jaeger et al. [5–7,10,11] took approximately 3–10 days on a 10-processor machine, for a total of approximately two years of CPU time for their study. In contrast, it took on the order of a day or two to optimize each of our models, on a single-processor machine running unoptimized, uncompiled MATLAB code. The relative speed of our technique was crucial to our study because it allowed us to rapidly explore alternative modeling formalisms and to test specific network structures. The strength of our technique (see Materials and Methods) lies in the approach it uses to produce initial estimates of the regulatory, decay, and diffusion parameters. With good initial estimates, the models can easily be fined-tuned by a straightforward search algorithm. Indeed, the simplicity of the procedures we used to solve each of the three stages speaks to the power of the decomposition. However, it is certainly possible to substitute other, more sophisticated methods for solving each stage. For example, Stage 2 comprises a set of function approximation problems, for which many fitting techniques are available. The optimization approach of Gursky et al. [12] is quite fast when given a good starting point, and it would be interesting to use their algorithm for Stage 3 of our approach.

#### Limitations of the models.

While our models, particularly Unc-GC, Unc-Logic, and the Combined model, capture the main features of gap gene expression dynamics, some failings are common to all the models. For example, none of the models capture well the shifting of the posterior *gt* domain (Figure 3). A gene circuit model based on the Sanchez and Thieffry regulatory relationships [4] (Protocol S1) does capture the shift, and by the expected mechanism, Hb repression of *gt*. However, the failure of the Unc-GC, Unc-Logic, and Combined models to capture the shift, despite including the necessary link, we can only attribute to imperfect optimization of the parameters. Our models also show defects in the early establishment of the domains, particularly the posterior *gt* domain (Figure 3C–3F, *t* = 11 min; similar defects were observed in [7]). This may be because the data is sampled less often early on, and so the RMS error effectively puts less weight on the correctness of the models early on. It may also be due to our simplification of transcription and translation into a single production process with no delay. At the start of cleavage cycle 13, gap gene transcripts begin to accumulate in the absence of gap proteins (except the Hb from maternally deposited mRNA). Thus, there should be no regulation between gap genes during the first few minutes of the time series. Our models allow regulation from the start of the time series, and this may be impacting the early expression patterns. Finally, none of the models capture the late parasegment 4-specific expression stripe of the anterior *hb* domain. This is visible as a small peak on top of the main anterior *hb* peak (Figure 1I or Figure 3B, *t* = 62 min). This expression stripe is due to a second *hb* promoter, different from the promoter responsible for the rest of the anterior *hb* domain [47]. It is likely that our gene circuit and logical models are simply incapable of capturing this phenomenon with a single production rate function, and would need to be generalized, perhaps by allowing *hb* production to be the sum of two separate production rate functions, to recreate the stripe.

Our models largely capture the wild-type expression patterns, and the regulatory relationships they rely on are consistent with mutant studies. However, our models do not in general display correct mutant expression patterns (see Protocol S6 for an example of simulating a *Kr* knockout mutant). The problem may point to a mismatch between biological reality and our modeling assumptions—for example, the mathematical forms of our production rate functions, our omission of production delays, or our treatment of protein concentrations as unitless numbers between 0 and 255. (Our expression data comes from images. Actual concentrations for the proteins are not known and may not even be of the same order of magnitude.) On the other hand, it is possible that the wild-type data to which the models were fit do not contain sufficient information to generalize to mutant organisms—although some gene circuit models fit to wild-type data do successfully predict mutant expression patterns [48]. Our fitting approach allows for rapid testing of alternative modeling assumptions, which may lead to models with improved predictions of mutant expression patterns. However, an important question for future research is whether we can find a way to incorporate the wealth of already available mutant expression data—which is often qualitative and not as temporally resolved as our wild-type data—into our fitting procedure.

Our models, particularly the Unc-GC model, includes a number of weak regulatory relationships, the significance of which is difficult to determine. Some of the links may arise from overfitting the data, or they may be compensating for incorrect modeling assumptions or for other missing or imperfectly modeled regulatory factors. However, it is difficult to say precisely which links should be ignored. Our data comprises a single space–time series, with strong correlations between datapoints. Resampling approaches for estimating significance, such as cross-validation or bootstrapping, are not useful in such cases. Jaeger et al. [7] deemed weights smaller in magnitude than a chosen threshold to be insignificant. However, the magnitudes of weights do not always reflect their importance to the model. For example, in the Unc-GC model, Tll activation of *hb* receives the smallest weight of any of *hb'*s regulators, and yet this link is crucial for the formation of the posterior *hb* domain. In contrast, some links that receive larger weights in the Unc-GC model are dispensible, as shown for example by the success of the RPJ-GC model at reproducing the *hb* expression patterns. For these reasons, we used stringent criteria for dismissing links based on modeling alone. (Gene circuit weights of magnitude less than 0.0001 and logical terms that have absolutely no effect on expression were discarded.) Instead, we turned to external validation, admitting links to our Combined model only if they are additionally supported by other experimental evidence. However, it is also possible that some of the weak links in the Unc-GC or other models are correct. Regulatory effects between the gap genes can be subtle. For example, some studies have found changes in *kni* expression in *gt* underexpression and overexpression conditions [22,35], while other studies did not detect any change [33,37]. Additional, careful quantitative measurements of gap gene expression in wild-type as well as mutant organisms will be necessary to resolve the existence of the weaker links in our models.

## Materials and Methods

### Quantitative gene expression data.

Quantitative gene expression data used in this study are available online in the FlyEx database http://urchin.spbcas.ru/flyex or http://flyex.ams.sunysb.edu/flyex [18]. Methods for data acquisition and processing are described in detail elsewhere [49–52]. The data include quantitative wild-type concentration profiles for the protein products of *bcd, cad, hb, Kr, kni, gt,* and *tll* during cleavage cycles 13 and 14A, which constitute the late syncytial blastoderm stage of *Drosophila* development [53,54]. This covers a time of approximately 70 min between the first unambiguous detection of gap protein [7] and the onset of gastrulation [54]. Expression data from cleavage cycle 12 are used for the initial concentration of Hb at *t* = 0. Initial concentrations of Kr, Kni, and Gt are zero. The data represent expression at ten times, two during cleavage cycle 13 (*t* = 0 and 11 min) and eight during cleavage cycle 14A (*t* = 24,30,37,43,49,55,62, and 68 min). These times differ slightly from the ones used in [7], because we use a fixed time step of one minute for simulating our models (see below). The exact data are available as Dataset S1.

### Model-fitting strategy.

Here we describe our three-stage approach to fitting the parameters of the partial differential equations. Details of how this strategy was applied for each model can be found in Protocol S2. The ultimate goal is to produce a partial differential equation model [12]:


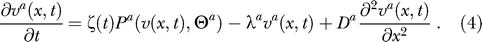


This is essentially the same as Equation 1, except that we have made explicit the regulatory parameters Θ*^a^* that need to be optimized. Θ*^a^* comprises *R^a^* and either the weights of a gene circuit model or thresholds of a logical model. The term *ζ*(*t*) models the doubling of nuclei and shutdown of transcription during mitosis as follows.


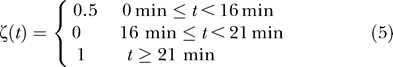


From the experience of Jaeger et al. [6,7], it would appear that direct optimization of the parameters, Θ*^a^, λ^a^,* and *D^a^* is difficult. The first two stages of our approach are intended to produce good initial estimates of these parameters. The third stage is a direct optimization approach that fine-tunes the initial estimates to produce a good fit to the data (see Figure 2 for a summary).

### Stage 1.

In the first stage, we estimate *λ^a^, D^a^* and the spatial and temporal extents of production associated with each domain. Peaks in gap protein expression are symmetrical, flat-topped, and have steeply sloping sides. It is reasonable to assume that, at any point in time, the rate of protein production associated with a particular peak is constant in some interval along the A–P axis and zero outside of that interval. However, it is not clear from the data exactly where such boundaries should be. It is also not clear exactly when production begins and when (or if) it ends.

We use a set of seven parameters to describe the conditions of protein production associated with each of the six gap protein peaks: *ρ,* a production rate; *τ_start_,* the time at which production begins; *τ_end_,* the time at which production ends; *x_s,a_,* the anterior-most extent of production at time *τ_start_; x_s,p_,* the posterior-most extent of production at time *τ_start_; x_e,a_,* the anterior-most extent of production at time *τ_end_;* and *x_e,p_,* the posterior-most extent of production at time *τ_end_*. The last six parameters define a quadrilateral region of space–time (Figure 2A). Within this quadrilateral, we take protein production to occur at rate *ρ*. Genes with multiple peaks are assigned one quadrilateral per peak. At space–time points that do not fall in any of the quadrilaterals for a particular protein, the rate of protein production is assumed to be zero. Thus, for a single-domain gene, the production rate function has the form


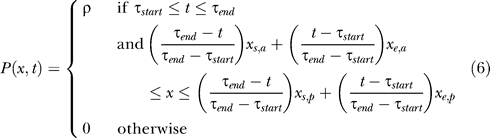


For a two-domain gene, the production rate function is the maximum of two such functions. In either case, the dynamical model of the expression of protein *a* is


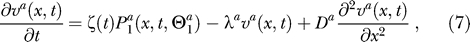


where 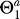 are parameters that specify the spatial extent of production and production rates for the expression domain or domains of protein *a*. To fully specify Equation 7 requires nine parameters for a single-domain gene and 16 parameters for a two-domain gene—a decay rate, a diffusion rate, and seven parameters describing the production conditions for each domain. The system of partial differential equations is decoupled—the genes do not interact—so the parameters for each gene can be optimized independently. We optimize the parameters for each gene to minimize the RMS error between simulated and observed expression, 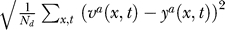 , where *v^a^*(*x*,*t*) is the simulated expression of protein *a*, *y^a^*(*x*,*t*), is the observed expression, and *N_d_* is the number of space–time points. We used a standard, repeated first-improvement local search with randomized order of neighbor examination to optimize the parameters for each gene. Stage 1 is performed only once for all four models, as it does not depend on the form of the *P^a^* or the regulatory structure or parameters.

### Stage 2.

Next, we generate an initial estimate of the regulatory parameters for each gene by searching for Θ^a^ that minimize the error function 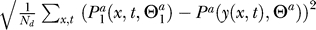 , where *N_d_* is the number of space–time points, 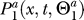 is the Stage 1 estimate for the production rate of protein *a* at space–time point (*x*,*t*), and *P^a^* is the production function for protein *a* (as in Equation 4, but with the observed expression values given as input). In other words, we search for regulatory parameters which fit, as closely as possible, the quadrilateral production regions found in Stage 1. Parameters can be optimized separately for each gene. This is just a problem in function approximation (also known as regression or supervised learning)—we seek a set of parameters Θ^a^ so that *P^a^* reproduces as well as possible the input–output pairs 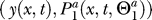 . Many techniques are available for solving this sort of problem. For our gene circuit models, the error function is differentiable with respect to Θ*^a^*. We optimize the parameters using repeated runs of an adaptive step-size gradient descent procedure. For our logical models, the regulatory parameters are optimized using a repeated first-improvement local search with randomized order of neighborhood examination.

Although the Unc-GC model includes Gt activation of *hb* and Kni activation of *gt,* we disallowed these links in the Unc-Logic model. There is no support in the literature for these links and we found that their removal improved our fits.

### Stage 3.

Finally, we combine the decay and diffusion constants estimated in Stage 1 with the regulatory parameters estimated in Stage 2 in a fully coupled partial differential equation model (Equation 4). Starting from these initial parameters, we perform repeated first-improvement local search with randomized order of neighbor examination, seeking parameters that minimize the RMS error 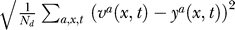 , where *N_d_* is the number of genes modeled (4) times the number of space–time points, *v^a^*(*x*,*t*) is the simulated expression from the model, and *y^a^*(*x*,*t*) is the observed expression.

### Numerical solution of the partial differential equations.

We simulate using a fixed time step of one minute and a spatial grid of 58 points (one space point for each 1% of embryo length between 35% and 92%). For each step, we calculate the production rates for each gene at each space point and add them to the expression values. This corresponds to one minute of constant-rate production with no decay or diffusion. For Equation 1 (or 4), in which production rates depend on the protein levels present, the simulated gap protein levels and the observed *bcd, cad,* and *tll* levels are used to calculate production. Values of *bcd, cad,* and *tll* are linearly interpolated in time for simulation times between the times for which we have observed values. We then calculate the result of one minute of decay and diffusion on the updated expression values. Equations 1 (or 4) and 7 can be solved analytically if there is no production. We assume reflecting boundary conditions 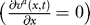 at the anterior boundary (the 35% line), because that boundary splits the anterior Hb and Gt peaks. Any protein diffusing out across the 35% line should be matched by protein diffusing in across the 35% line from the parts of the peaks not modeled. At the posterior boundary (the 92% line), we use an absorbing boundary condition (*v^a^*(*x*,*t*) = 0), because that boundary does not intersect any of the gap gene peaks.

## Supporting Information

Dataset S1 Observed Expression Data (41 KB DOC)

Protocol S1 Fits Based on the Sanchez–Thieffry Network Structure (58 KB PDF)

Protocol S2 Optimization Method Details (75 KB PDF)

Protocol S3 Estimated Parameter Values (10 KB PDF)

Protocol S4 Fits Based on Combined Structure Network (56 KB PDF)

Protocol S5 Comparison of Errors in the Jaeger et al. Model and Unc-GC (38 KB PDF)

Protocol S6 Simulating a Mutant *Kr*- Embryo (59 KB PDF)

## Abbreviations

A–P: anterior–posterior
Bcd: Bicoid
*bcd*: *bicoid*
Cad: Caudal
*cad*: *caudal*
*gt*: *giant*
*Hb*: *hunchback*
*kni*: *knirps*
*Kr*: *Krüppel*
RPJ network structure: Rivera-Pomar and Jäckle network structure
Tll: tailless
*tll*: *tailless*
