# Supplementary material for: Reverse Engineering the Gap Gene Network of Drosophila melanogaster
Source: PLoS Comput Biol. 2006 May 19;2(5):e51. doi: 10.1371/journal.pcbi.0020051 (PMC1463021; doi:10.1371/journal.pcbi.0020051)
Supplement: Supplementary file 1 [file pcbi.0020051.sd001.pdf]

Protocol S1. Fits Based on the Sanchez-Thieffry Network Structure

We fit models using the regulatory relationships in the model of Sanchez and Thieffry [1], with the addition of Tll activating *hb*. This link was necessary to activate the posterior *hb* domain, just as in all of our other models. We call this set of regulatory relationships the ST regulatory relationships. The best-fitting gene circuit model using these regulatory relationships, ST-GC, had an RMS error of 14.63, while our best-fitting ST-Logic model had an RMS error of 22.20. With the exception of Hb activation of *Kr*, the ST regulatory relationships are a subset of those in the Combined model, and with the exception of Hb repression of *gt*, they are a subset of the RPJ relationships. Like our RPJ-GC model, the ST-GC model fails to repress *Kr* in the anterior of the trunk at the early time points (Figure 2D11,24), though the ST-GC model does not display the same degree of ectopic *Kr* expression in the posterior as the RPJ-GC model does. Like the RPJ-Logic model, the ST-Logic model fails to sustain its posterior *gt* domain (Figure 2E). Thus, the ST models share the two major failings of the RPJ models. However, the ST-GC model does a better job of capturing the shifting of the posterior *gt* domain than any of our other models, including Unc-GC (Figure 2D). It does so by employing strong repression from Hb (Figure 1), a feature that was not found in any of our other models, but that was found by Jaeger et al. [2, 3].

ST-GC

Gene	Max prod. rate (R^a)	regulatory weights (T^{ab}) Bcd	Cad	Hb	$\mathrm{Hb}^2/255$	Kr	Gt	Kni	Tll	Bias (h^a)	Decay (λ^a)	Diff. (D^a)
Hb	36.5801	0.1499	·	0.0314	·	-0.0272	·	·	0.0172	-3.5	0.1522	2.827
Kr	13.8279	0.9549	·	0.2054	-0.6921	·	-0.3255	-0.0629	·	-3.5	0.0637	0.463
Gt	9.4553	1.7412	*0.0001*	-0.1735	·	-0.6990	·	·	·	-3.5	0.0615	0.116
Kni	9.2782	0.0195	0.0790	-0.1613	·	·	-0.5268	·	·	-3.5	0.0285	0.118

ST-Logic

Gene	Max prod. rate (R^a)	Production Rule	Decay (λ^a)	Diff. (D^a)
Hb	30.5	(Bcd≥20 or Hb≥59 or Tll≥124) and Kr≤148	0.139	1.54
Kr	16.5	(Bcd≥6 or *Hb≥146*) and Hb≤154 and Gt≤4 and Kni≤97	0.066	0.7
Gt	18	(Bcd≥39 or Cad≥130) and *Hb≤208* and Kr≤15	0.062	0.43
Kni	17.4	(Bcd≥6 or Cad≥139) and Hb≤4 and *Gt≤114*	0.073	0.73

Figure 1: Parameters for best-scoring models using the ST regulatory relationships, with gene circuit and logical formalisms for production rate functions.

References

[1] Sanchez L, Thieffry D (2001) A logical analysis of the gap gene system. Journal of Theoretical Biology 211:115–141.

[2] Jaeger J, Surkova S, Blagov M, Janssens H, Kosman D, et al. (2004) Dynamic control of positional information in the early *Drosophila* embryo. Nature 430:368–371.

[3] Jaeger J, Blagov M, Kosman D, Kozlov KN, Manu, et al. (2004) Dynamical analysis of regulatory interactions in the gap gene system of *Drosophila melanogaster*. Genetics 167:1721–1737.

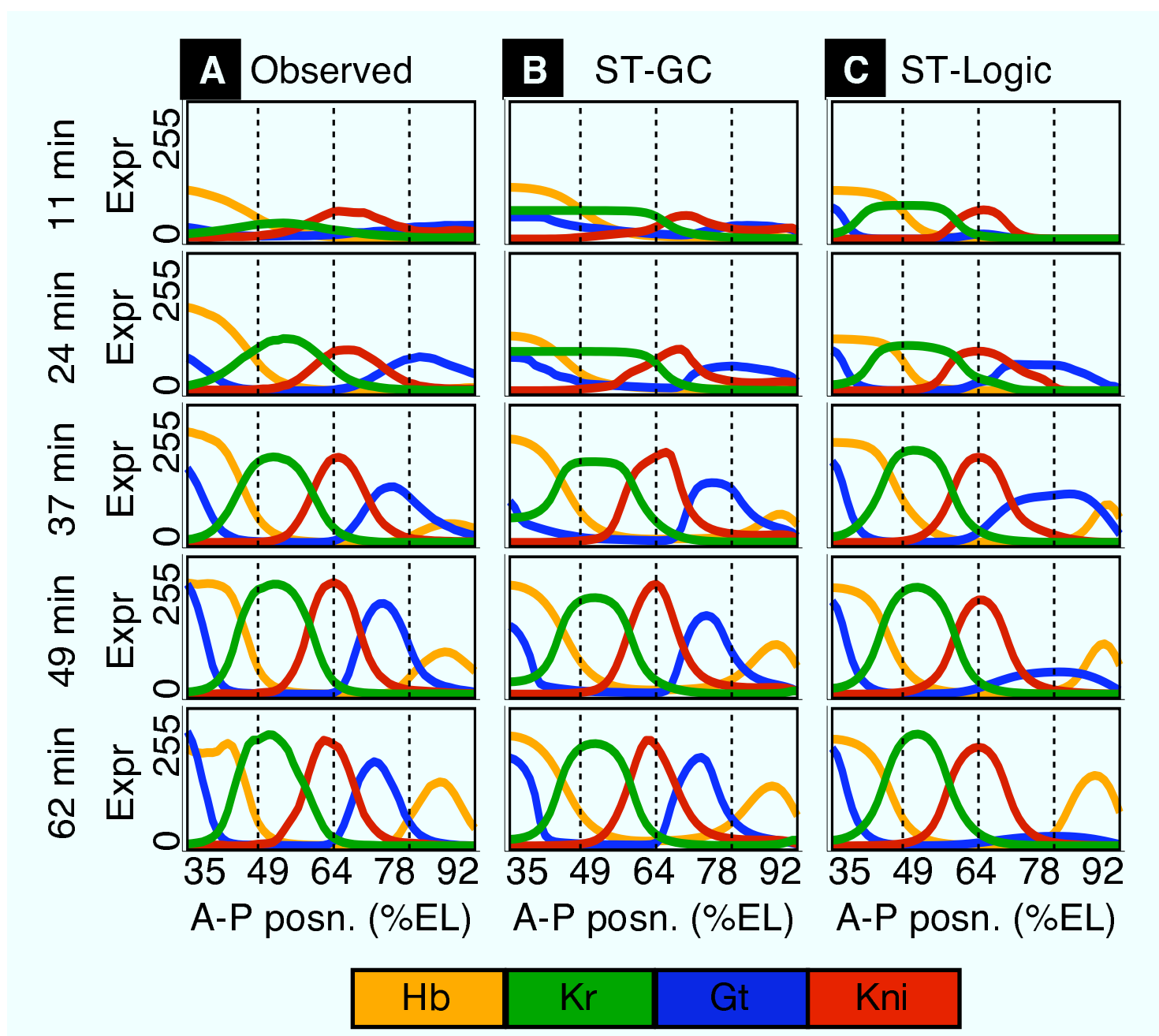

Figure 2: Observed gap expression and simulated expression from the ST models.

.
