# Supplementary material for: Reverse Engineering the Gap Gene Network of Drosophila melanogaster
Source: PLoS Comput Biol. 2006 May 19;2(5):e51. doi: 10.1371/journal.pcbi.0020051 (PMC1463021; doi:10.1371/journal.pcbi.0020051)
Supplement: Supplementary file 2 [file pcbi.0020051.sd002.pdf]

Protocol S2. Optimization Method Details

Stage 1

Recall that Stage 1 produces initial estimates of the decay (λ^a) and diffusion (D^a) parameters of each gene, and parameters describing the rate and spatial and temporal extents of production associated which each expression domain (7 parameters):

ρ	production rate
τ_{start}	start time of production (min)
τ_{end}	end time of production (min)
$x_{s,a}$	anterior-most extent of production at time τ_{start} (% EL=embryo length)
$x_{s,p}$	posterior-most extent of production at time τ_{start} (% EL)
$x_{e,a}$	the anterior-most extent of production at time τ_{end} (% EL)
$x_{e,p}$	the posterior-most extent of production at time τ_{end} (% EL)

Parameters for each gene are optimized independently, using 100 runs of a repeated first-improvement local search with randomized order of examination. Each local search run comprises a sequence of steps through parameter space, starting from initial estimates which we constructed based on manual analysis of the expression data:

Gene	(domain)	ρ	τ_{start}	τ_{end}	$x_{s,a}$	$x_{s,p}$	$x_{e,a}$	$x_{e,p}$	λ^a	D^a
hb	(anterior)	15.6	0	68	35	46	35	46	0.075	1.00
	(posterior)	8.9	30	68	79	89	79	89		
Kr		15.7	0	68	44	58	44	58	0.075	1.00
gt	(anterior)	15.9	0	68	35	38	35	38	0.075	1.00
	(posterior)	12.6	0	68	67	76	67	76		
kni		15.6	0	68	57	67	57	67	0.075	1.00

At each step of the local search, "neighboring" parameter sets are examined in random order, looking for one that produces simulated expression with lower root mean squared error compared to the observed data. The first neighboring parameter set found with lower error is adopted, and search continues from there. A neighboring parameter set is one in which a single parameter is changed by an amount: ± 0.1 for ρ, ± 1 for τ_{start} for posterior *hb* only (all other start and stop times were held fixed), ± 1 for the space parameters except for $x_{s,a}$ and $x_{e,a}$ for anterior *hb* and *gt* (because we know those domains are split by the 35% line), ± 0.001 for λ^a, and ± 0.01 for D^a. The local search continues until it reaches a locally optimal parameter set—one for which no neighbor has lower root mean squared error. The best parameter set found is shown below.

Gene	(domain)	ρ	τ_{start}	τ_{end}	$x_{s,a}$	$x_{s,p}$	$x_{e,a}$	$x_{e,p}$	λ^a	D^a
hb	(anterior)	30.1	0	68	35	48	35	46	0.139	1.08
	(posterior)	16.9	35	68	82	101	79	88		
Kr		15.5	0	68	44	64	45	56	0.065	0.60
gt	(anterior)	16.0	0	68	35	37	35	38	0.062	0.40
	(posterior)	11.2	0	68	74	89	66	73		
kni		15.9	0	68	60	74	58	66	0.065	0.60

Stage 2

For each gap protein a, the best-scoring parameter set from Stage 1, Θ_1^a, provides an estimated production rate $P_1^a(x,t,\Theta_1^a)$, for each space x and time t. For example, the rule above states that Hb is produced at rate 30.1 if (x,t) falls within the first quadrilateral, rate 16.9 if (x,t) falls within the second quadrilateral, and rate 0 otherwise. Stage 2 makes an initial estimate of the regulatory parameters for gene a, Θ^a (see Equation 2 in the paper), by searching for parameters that minimize the root mean squared error $\sqrt{\frac{1}{N_d}\sum_{x,t}(P_1^a(x,t,\Theta_1^a) - P^a(y(x,t),\Theta^a))^2}$, where $y(x,t)$ is the vector of observed expression values of all proteins at space x and time t, and N_d is the number of space-time points in the data set.

To optimize parameters for the gene circuit models, we performed 100 runs of 10000 steps of gradient-descent optimization of the production rate (R^a) and the regulatory weights (T^{ab}). Optimization for each gene is independent. We used an adaptive step size for the gradient descent. The step size was initialized to 10^{-3} and increased by a factor of 1.01 for every step that decreased the error. Any step that increased the error was retracted and the step size reduced by one half. Each R^a was initialized to the maximum value of P_1^a. The T^{ab} were initialized independently randomly in the interval $[-0.1,+0.1]$ for each unconstrained fitting run. For the network constrained to follow the RPJ structure, weights were initialized in the range $[-0.1,0]$ or $[0,+0.1]$, depending on whether they represent repression or activation respectively. The correct sign of weights was maintained by setting a weight to zero any time a gradient step would change its sign.

For the logical models, the repression threshold for a protein b acting on a gene a was set to $\max_{\{(x,t):P_1^a(x,t)>0\}} v^b(x,t)$. This creates repression at as many space-time points as possible while ensuring there is no repression at space-time points estimated in Stage 1 to have production, except for point(s) right at the threshold. Activation thresholds were optimized by 100 runs of first-improvement local search with randomized order of neighborhood examination. Neighboring parameter sets comprised all single changes of activation thresholds by an amount $\pm 2^z$ for $z \in \{0,1,2,\ldots,5\}$. At the start of each run, the activation threshold for a protein b acting on a gene a was intialized uniformly randomly between the minimum and maximum observed values of v^b. The production rates for each gene were set to the maximum rate estimated for the gene in Stage 1.

Best-fitting parameters for each model are shown in Figure 1. Many parameters are similar to the final values obtained after Stage 3 (see Supplementary Information S1). However, simulating these models without further optimization gives a poor fit to the data (Figure 2).

Stage 3

In Stage 3, the parameters obtained in Stages 1 and 2 are put into a coupled partial differential equation model (all four trunk gap genes) and optimized by direct search. That is, a parameter set is evaluated by computing the solution to the partial differential equations and then by computing the root mean squared error between simulated and observed expression.

For the gene circuit models, we used 10 runs of first-improvement local search with random order of neighborhood examination to optimize the parameters (each run starting from the best set of regulatory parameters found in Stage 2 and the best decay and diffusion parameters found in Stage 1). We initially experimented with a neighborhood consisting of perturbations of single variables: ± 0.0001 for the λ^a,

Unc-GC

Gene	Max prod. rate (R^a)	regulatory weights (T^{ab}) Bcd	Cad	Hb	Kr	Gt	Kni	Tll	Bias (h^a)	Decay (λ^a)	Diff. (D^a)
Hb	30.09	0.1417	-0.0045	0.0365	-0.0189	0.0192	-0.0563	0.0127	-3.5000	0.139	1.08
Kr	15.51	0.1246	0.0214	-0.0465	0.0687	0.0063	-0.0746	-0.0891	-3.5000	0.065	0.60
Gt	16.00	0.0626	0.0174	0.0005	-0.0465	0.0156	0.0051	-0.0126	-3.5000	0.062	0.40
Kni	15.91	0.2005	0.0208	-0.1873	-0.0492	-0.1461	0.0837	-0.1799	-3.5000	0.065	0.60

Unc-Logic

Gene	Max prod. rate (R^a)	Production Rule	Decay (λ^a)	Diff. (D^a)
Hb	30.1	(Bcd≥23 or Hb≥65 or Tll≥139) and Cad≤117 and Kr≤170 and Kni≤7	0.139	1.08
Kr	15.5	(Bcd≥10 or Cad≥151 or Kr≥103) and Hb≤169 and Gt≤8 and Kni≤126 and Tll≤6	0.065	0.6
Gt	16	(Bcd≥39 or Cad≥135 or Gt≥91) and Hb≤208 and Kr≤16 and Tll≤33	0.062	0.4
Kni	15.9	(Bcd≥5 or Cad≥152 or Kni≥92) and Hb≤8 and Kr≤148 and Gt≤88 and Tll≤6	0.065	0.6

RPJ-GC

Gene	Max prod. rate (R^a)	regulatory weights (T^{ab}) Bcd	Cad	Hb	$Hb^2/255$	Kr	Gt	Kni	Tll	Bias (h^a)	Decay (λ^a)	Diff. (D^a)
Hb	30.0519	0.1222	·	0.0340	·	-0.0160	·	·	0.0116	-3.5	0.1390	1.080
Kr	14.9554	0.4780	·	0.0000	-0.0757	·	-0.4347	-0.0192	-0.0213	-3.5	0.0650	0.600
Gt	15.7765	0.1312	0.0310	·	·	-0.1829	·	-0.0000	-0.0364	-3.5	0.0620	0.400
Kni	15.8249	0.4260	0.0173	-0.9033	·	0.0000	-0.0105	·	-0.2074	-3.5	0.0650	0.600

RPJ-Logic

Gene	Max prod. rate (R^a)	Production Rule	Decay (λ^a)	Diff. (D^a)
Hb	30.1	(Bcd≥23 or Hb≥65 or Tll≥140) and Kr≤170	0.139	1.08
Kr	15.5	(Bcd≥10 or Hb≥4) and Hb≤169 and Gt≤8 and Kni≤126 and Tll≤6	0.065	0.6
Gt	16	(Bcd≥38 or Cad≥137) and Kr≤16 and Kni≤156 and Tll≤33	0.062	0.4
Kni	15.9	(Bcd≥5 or Cad≥175 or Kr≥187) and Hb≤8 and Gt≤88 and Tll≤6	0.065	0.6

Figure 1: The best-scoring regulatory parameters from Stage 2, along with the decay and diffusion rates from Stage 1. Together, these comprise our initial estimates of all model parameters.

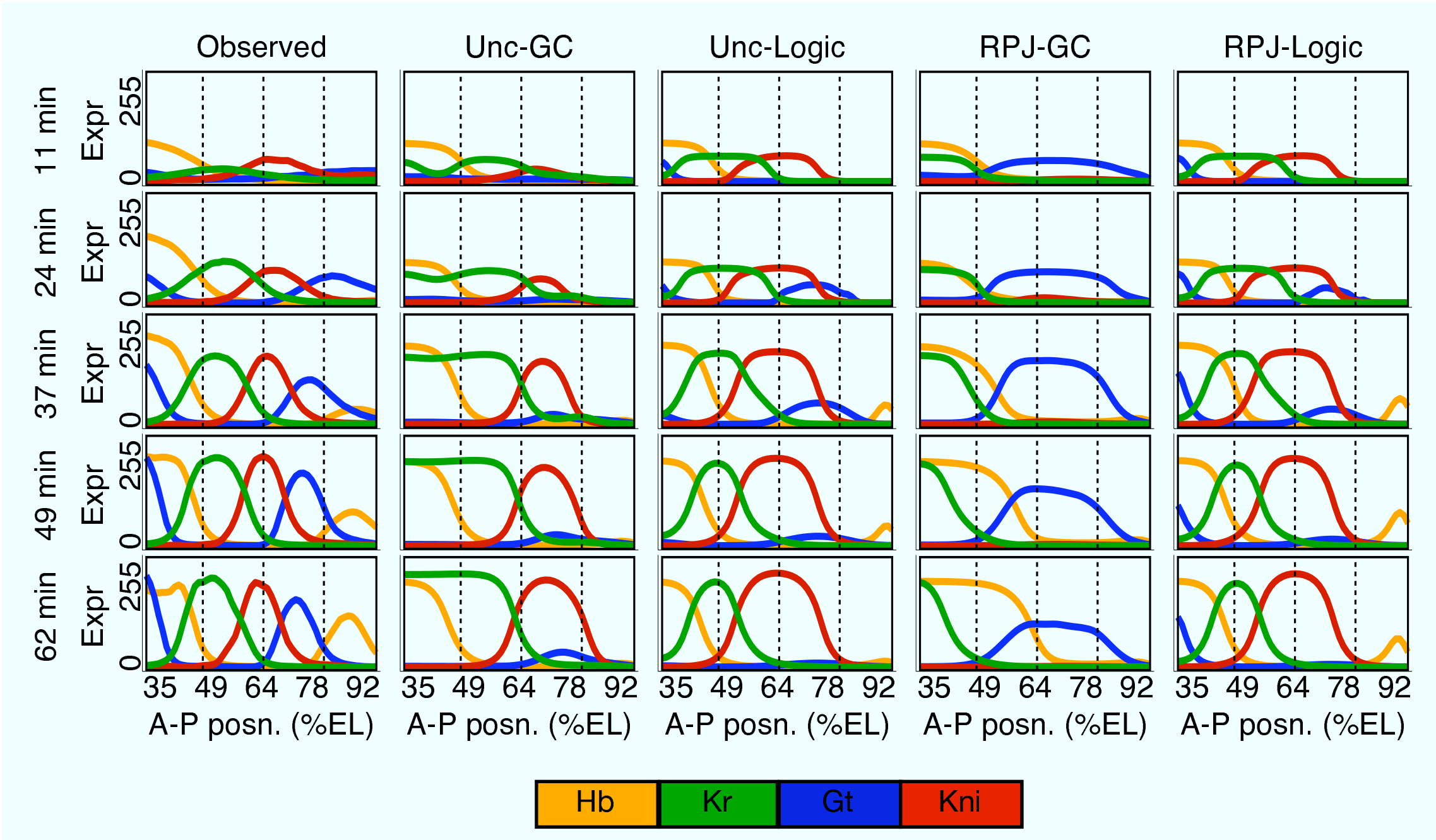

Figure 2: Observed and simulated expression of the gap proteins from the models after the first two stages of optimization. Model parameters are shown in Figure 1.

.

± 0.0001 for the D^a, ± 0.01 for the R^a, and ± 0.0001 for the T^{ab}. We found that most of the parameters changed little, but a few changed significantly in magnitude, requiring many local search steps. Thus, we adopted an adaptive step-size for the local search. Each time a perturbation resulted in a lower RMS error between simulated and observed expression, that perturbation was accepted and the magnitude of the step-size for that parameter was doubled. Every time neither an increase nor a decrease in a parameter at its current step-size produced an improvement in the error, the step-size was set to half its current value or its initial value, whichever was larger. A search run terminated when all step-sizes were at their starting (minimal) value and no neighboring solutions had smaller RMS error than the current solution.

For the logical models, we used 50 runs of first-improvement local search with random order of neighborhood examination. We allowed more runs for the logical models because the average duration of one run was much smaller than for the gene circuit models. The neighborhood comprised single perturbations of ± 0.001, ± 0.002, ± 0.005, ± 0.01 or ± 0.02 for the λ^a; ± 0.01, ± 0.02, ± 0.05, ± 0.1 or ± 0.2 for the D^a; ± 0.1, ± 0.2, ± 0.5, ± 1 or ± 2 for the R^a; and ± 1, ± 2, ± 4, ± 8 or ± 16 for the activation and repression thresholds.
