# Supplementary material for: Reverse Engineering the Gap Gene Network of Drosophila melanogaster
Source: PLoS Comput Biol. 2006 May 19;2(5):e51. doi: 10.1371/journal.pcbi.0020051 (PMC1463021; doi:10.1371/journal.pcbi.0020051)
Supplement: Supplementary file 3 [file pcbi.0020051.sd003.pdf]

Protocol S3. Estimated Parameter Values

Estimated parameter values for the best-scoring Unc-GC, Unc-Logic, RPJ-GC and RPJ-Logic models are shown below. Parameters in italics are considered to have been eliminated by optimization. Regulatory weights in gene circuit models are considered eliminated if the weight is less than or equal to 0.0001 in absolute value. Terms in the logical models are considered eliminated if removing them from the rule results in no change in simulated expression. The production and decay rates have units min^{-1}, and the diffusion rates have units (1% embryo length)^2 min^{-1}. To obtain the diffusion rates in the more traditional units of $\text{cm}^2\text{sec}^{-1}$, one should multiply by 1.5×10^{-9}. This assumes an embryo 0.3mm long.

Unc-GC

Gene	Max prod. rate (R^a)	regulatory weights (T^{ab}) Bcd	Cad	Hb	Kr	Gt	Kni	Tll	Bias (h^a)	Decay (λ^a)	Diff. (D^a)
Hb	32.03	0.1114	−0.0054	0.0293	−0.0124	0.0553	−0.3903	0.0144	−3.5	0.136	2.25
Kr	16.70	0.1173	0.0215	−0.0498	0.0755	−0.0141	−0.0666	−1.2036	−3.5	0.072	0.55
Gt	25.15	0.0738	0.0180	−0.0008	−0.0758	0.0157	0.0056	−0.0031	−3.5	0.109	0.17
Kni	16.12	0.2146	0.0210	−0.1891	−0.0458	−0.1458	0.0887	−0.3028	−3.5	0.065	0.51

Unc-Logic

Gene	Max prod. rate (R^a)	Production Rule	Decay (λ^a)	Diff. (D^a)
Hb	31.3	(Bcd≥19 or Hb≥49 or Tll≥121) and Cad≤122 and Kr≤139 and Kni≤4	0.146	2.16
Kr	16.1	(Bcd≥9 or *Cad≥151* or *Kr≥103*) and *Hb≤169* and Gt≤4 and Kni≤115 and *Tll≤20*	0.067	0.67
Gt	16.3	(Bcd≥35 or Cad≥133 or Gt≥85) and *Hb≤208* and Kr≤15 and *Tll≤102*	0.087	0.4
Kni	17	(Bcd≥5 or *Cad≥152* or *Kni≥94*) and Hb≤7 and *Kr≤141* and Gt≤70 and *Tll≤18*	0.067	0.9

RPJ-GC

Gene	Max prod. rate (R^a)	regulatory weights (T^{ab}) Bcd	Cad	Hb	$\text{Hb}^2/255$	Kr	Gt	Kni	Tll	Bias (h^a)	Decay (λ^a)	Diff. (D^a)
Hb	41.0319	0.1678	·	0.0317	·	−0.0342	·	·	0.0159	−3.5	0.1650	2.963
Kr	12.6054	0.7879	·	0.3923	−1.0593	·	−0.1644	−0.0775	−0.1767	−3.5	0.0547	0.421
Gt	10.3065	0.6292	0.0277	·	·	−0.3716	·	*−0.0001*	−0.0964	−3.5	0.0670	0.122
Kni	7.9349	*0.0000*	0.1222	−0.4368	·	*0.0001*	−0.5568	·	−2.9999	−3.5	0.0135	0.130

RPJ-Logic

Gene	Max prod. rate (R^a)	Production Rule	Decay (λ^a)	Diff. (D^a)
Hb	30.7	(Bcd≥23 or Hb≥58 or Tll≥125) and Kr≤148	0.141	1.65
Kr	16.4	(Bcd≥6 or *Hb≥1*) and Hb≤149 and Gt≤8 and Kni≤123 and Tll≤4	0.071	0.67
Gt	18	(Bcd≥38 or Cad≥128) and Kr≤16 and *Kni≤156* and Tll≤69	0.062	0.5
Kni	17.1	(Bcd≥6 or *Cad≥175* or *Kr≥187*) and Hb≤4 and *Gt≤109* and *Tll≤6*	0.073	0.85
