# Supplementary material for: Reverse Engineering the Gap Gene Network of Drosophila melanogaster
Source: PLoS Comput Biol. 2006 May 19;2(5):e51. doi: 10.1371/journal.pcbi.0020051 (PMC1463021; doi:10.1371/journal.pcbi.0020051)
Supplement: Supplementary file 4 [file pcbi.0020051.sd004.pdf]

Protocol S4. Fits Based on the Combined Network Structure

The regulatory relationships in the Combined model are sufficient to fit the expression data using either the gene circuit formalism (RMS error 14.01) or the logical formalism (RMS error 14.91) for representing the production rate functions. Figure 1 shows the parameters from the best-scoring fits. The simulated expression resulting from these models is shown in Figure 2B,C.

Combined-GC

	Max prod.	regulatory weights (T^{ab})							Bias	Decay	Diff.
Gene	rate (R^a)	Bcd	Cad	Hb	Kr	Gt	Kni	Tll	(h^a)	(λ^a)	(D^a)
Hb	32.5274	0.1090	·	0.0332	-0.0220	·	-0.0303	0.0179	-3.5	0.1377	1.997
Kr	17.0549	0.4517	·	-0.2671	0.2506	-0.0413	-0.2549	*-0.0001*	-3.5	0.0647	0.918
Gt	22.6812	0.0691	0.0180	-0.0023	-0.0235	0.0185	·	-0.0055	-3.5	0.1125	0.148
Kni	13.0381	0.6958	0.0204	-1.1855	·	-0.0049	·	-0.3178	-3.5	0.0381	0.269

Combined-Logic

Gene	Max prod. rate (R^a)	Production Rule	Decay (λ^a)	Diff. (D^a)
Hb	30.6	(Bcd≥23 or Hb≥54 or Tll≥126) and Kr≤148 and Kni≤3	0.146	1.48
Kr	15.5	(Bcd≥9 or *Kr≥147*) and Hb≤147 and Gt≤7 and Kni≤123 and Tll≤4	0.065	0.61
Gt	16	(Bcd≥38 or Cad≥132 or Gt≥91) and *Hb≤208* and Kr≤17 and Tll≤94	0.076	0.64
Kni	16.7	(Bcd≥5 or *Cad≥175*) and Hb≤3 and Gt≤80 and *Tll≤8*	0.07	0.74

Figure 1: Parameters for best-scoring models using the Combined regulatory relationships, with gene circuit and logical formalisms for production rate functions.

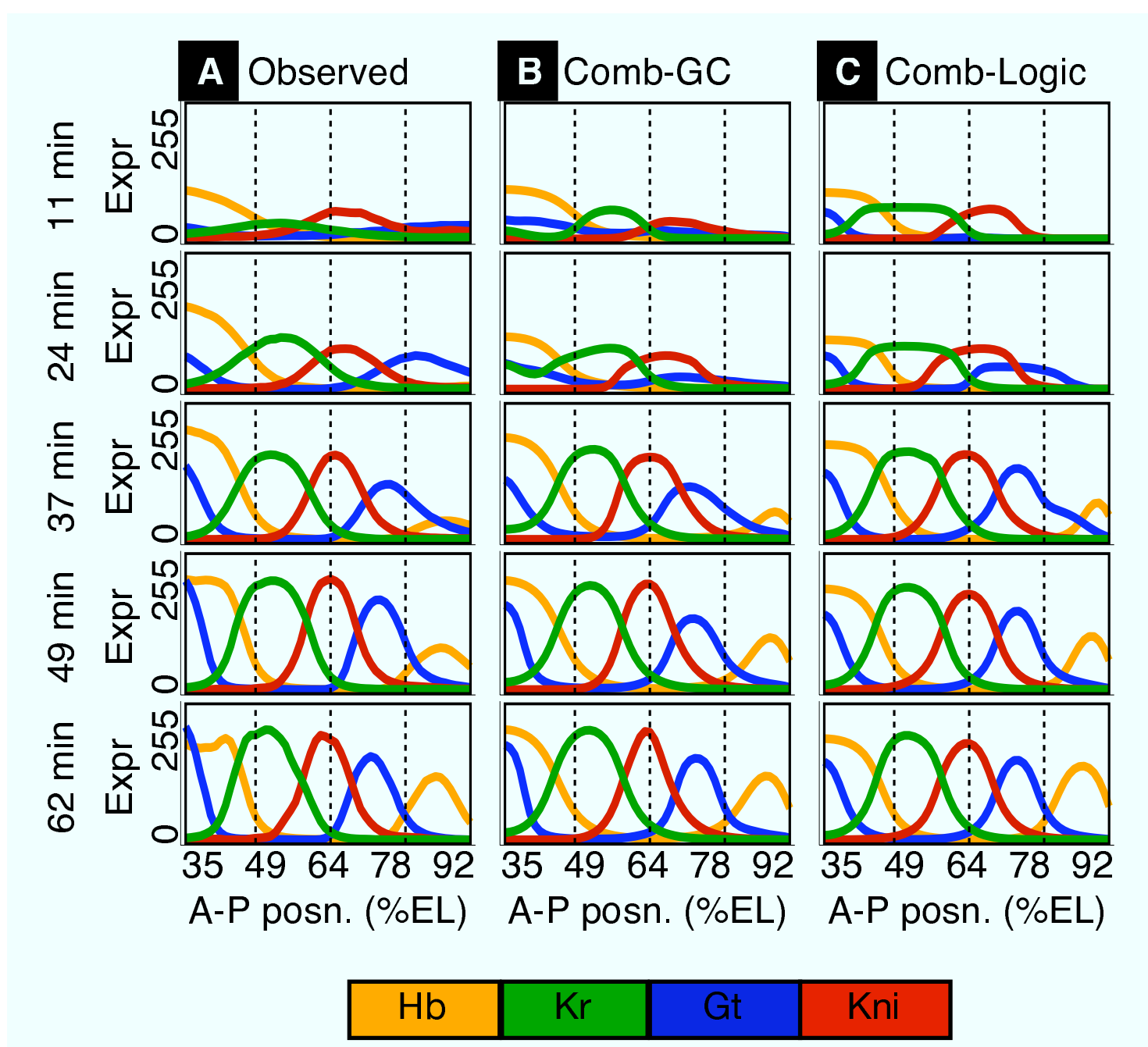

Figure 2: Observed gap expression and simulated expression from the Combined models.

.
