# Supplementary material for: Reverse Engineering the Gap Gene Network of Drosophila melanogaster
Source: PLoS Comput Biol. 2006 May 19;2(5):e51. doi: 10.1371/journal.pcbi.0020051 (PMC1463021; doi:10.1371/journal.pcbi.0020051)
Supplement: Supplementary file 5 [file pcbi.0020051.sd005.pdf]

Protocol S5. Comparison of Errors in the Jaeger et al. Model and Unc-GC

The RMS error of our Unc-GC model is 12.29. Jaeger et al. [1] reported an RMS error of 9.420 for their best-fitting model. However, these numbers are not directly comparable. Jaeger et al. model the dynamics of *cad* and *tll* expression in addition to that of the trunk gap genes, treating only *bcd* as an exogenous input. Their RMS error is thus computed over all six genes: *cad*, *hb*, *Kr*, *gt*, *kni*, *tll*. The RMS error of their best model computed over just the trunk gap genes is 12.08. This is lower than the error of Unc-GC, but the difference is small—much smaller than the differences between any of our four models—and well within the experimental error in the data. Figure 1 shows overlaid plots of the observed data, simulated expession according to the Jaeger et. al model, and simulated expression according to Unc-GC. The Jaeger et al. model does slightly better at capturing *gt* expression early on. Unc-GC captures posterior *hb* better. But even these differences are not dramatic, and the fits are otherwise very similar. We do not consider either model to be a significantly better fit to the data than the other.

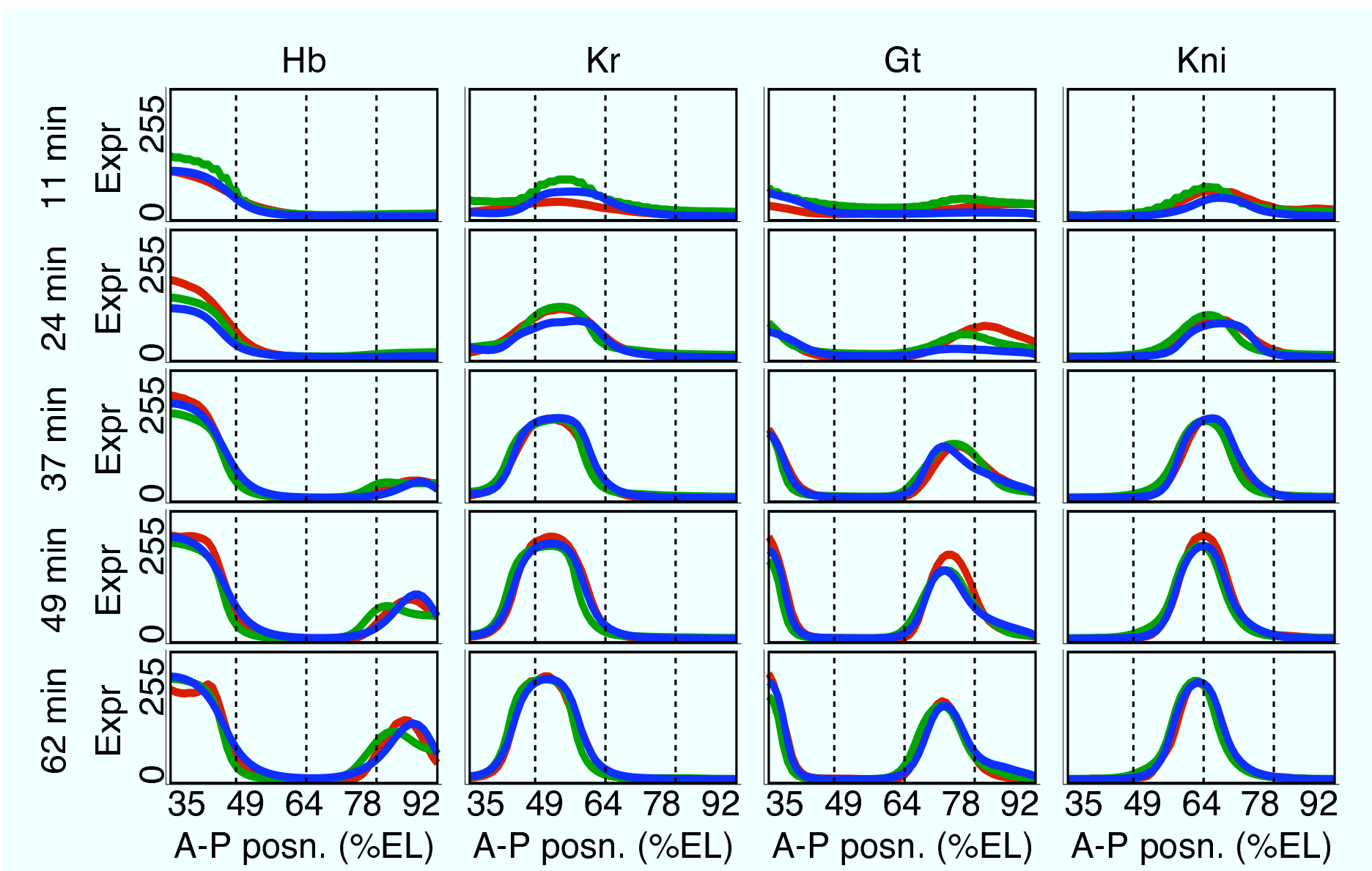

Figure 1: Comparison of observed trunk gap gene expression (red), simulated expression according to the best-fitting model of Jaeger et al. [1] (green), and simulated epxression of Unc-GC.

References

[1] Jaeger J, Blagov M, Kosman D, Kozlov KN, Manu, et al. (2004) Dynamical analysis of regulatory interactions in the gap gene system of *Drosophila melanogaster*. Genetics 167:1721–1737.
