# Supplementary material for: Reverse Engineering the Gap Gene Network of Drosophila melanogaster
Source: PLoS Comput Biol. 2006 May 19;2(5):e51. doi: 10.1371/journal.pcbi.0020051 (PMC1463021; doi:10.1371/journal.pcbi.0020051)
Supplement: Supplementary file 6 [file pcbi.0020051.sd006.pdf]

Protocol S6. Simulating a Mutant *Kr*- Embryo

In general, our models do not correctly reproduce mutant expression patterns, despite the fact the the regulatory relationships in our models are qualitatively consistent with mutant observations. For example, Figures 1 and 2 show the predicted expression patterns for a *Kr*- mutant according to the Unc-GC and Unc-Logic models. These predictions are obtained by simulating the models from the standard initial conditions, but fixing Kr production to zero. We expect, of course, that *Kr* is not expressed, as seen in the models. *hb*, which is redundantly repressed between its two usual domains by Kr and Kni, should show little or no change. At most, a slight expansion of the anterior *hb* domain towards the posterior should be observed [1]. The Unc-Logic prediction, then, is essentially correct. However, the Unc-GC model incorrectly shows strong derepression of *hb* between its two usual domains. Roughly speaking, the Unc-GC model encodes a rule that both Kr and Kni are required for repression of *hb*, whereas the real organism behaves as if either Kr or Kni are individually sufficient for repression [1, 2]. In principle, the Unc-GC model is able to encode either rule. Because the mutant data was not part of the fitting processes, it is perhaps quite arbitrary that the fitting procedure settled on the former rule instead of the latter—either is a reasonable extrapolation from the wild-type data. *gt* should show some derepression between its two domains, with both domains expanding towards each other but not meeting [3, 4]. Again, the Unc-GC model overestimates the effect of removing repression from Kr. The Unc-Logic model shows no change in *gt* expression, underestimating the true effect. Expression of *kni* is reduced in *Kr*- embryos, due to broadening of the *gt* domains [3, 4, 5, 6]. Unc-Logic, because it predicts no change in *gt* expression, does not capture this effect, while the Unc-GC model, which overestimates the change in *gt* expression, completely quashes *kni* expression.

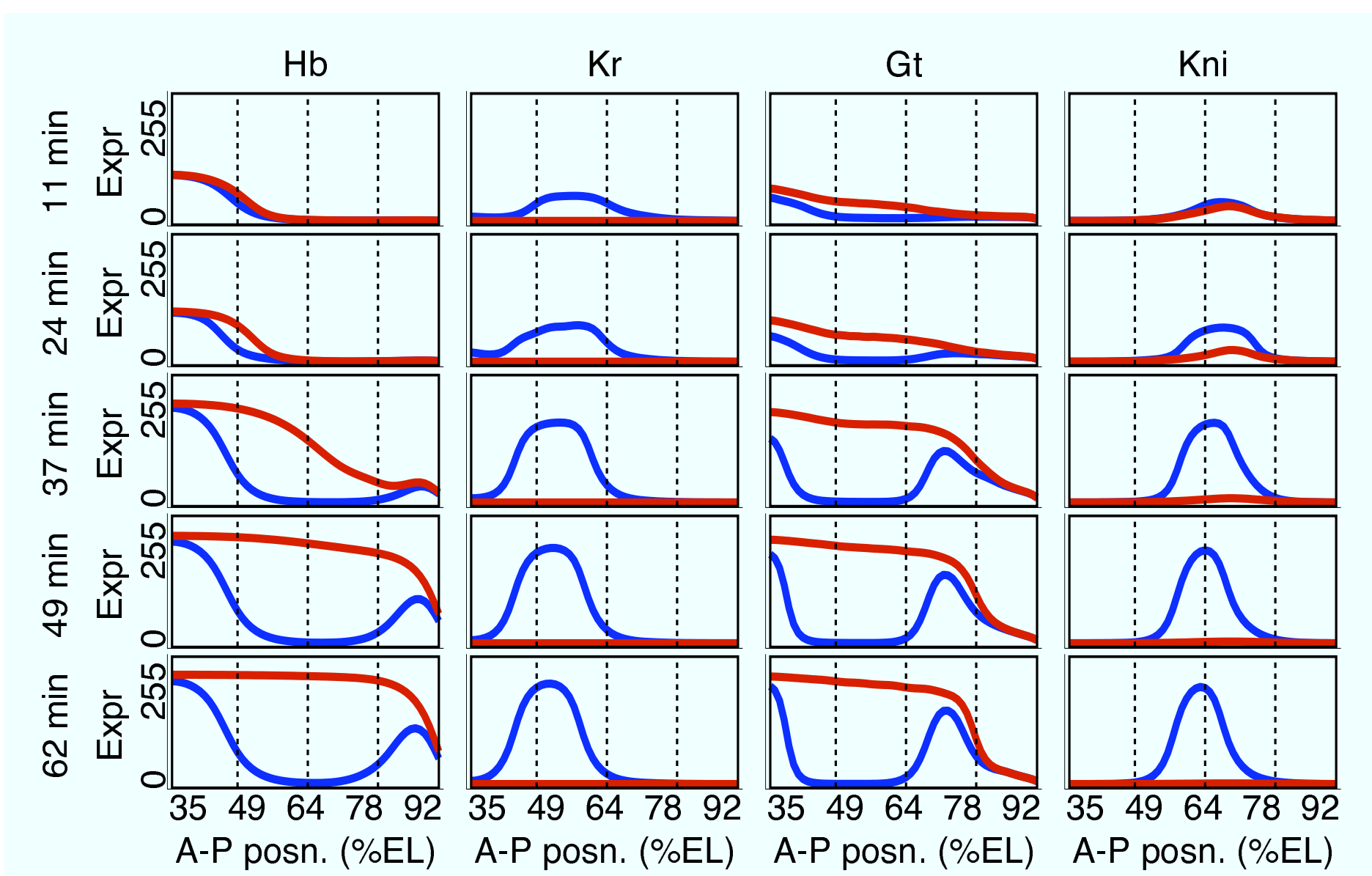

Figure 1: Simulated wild-type expression patterns from the Unc-GC model (blue) and expression patterns predicted for a *Kr*- embryo.

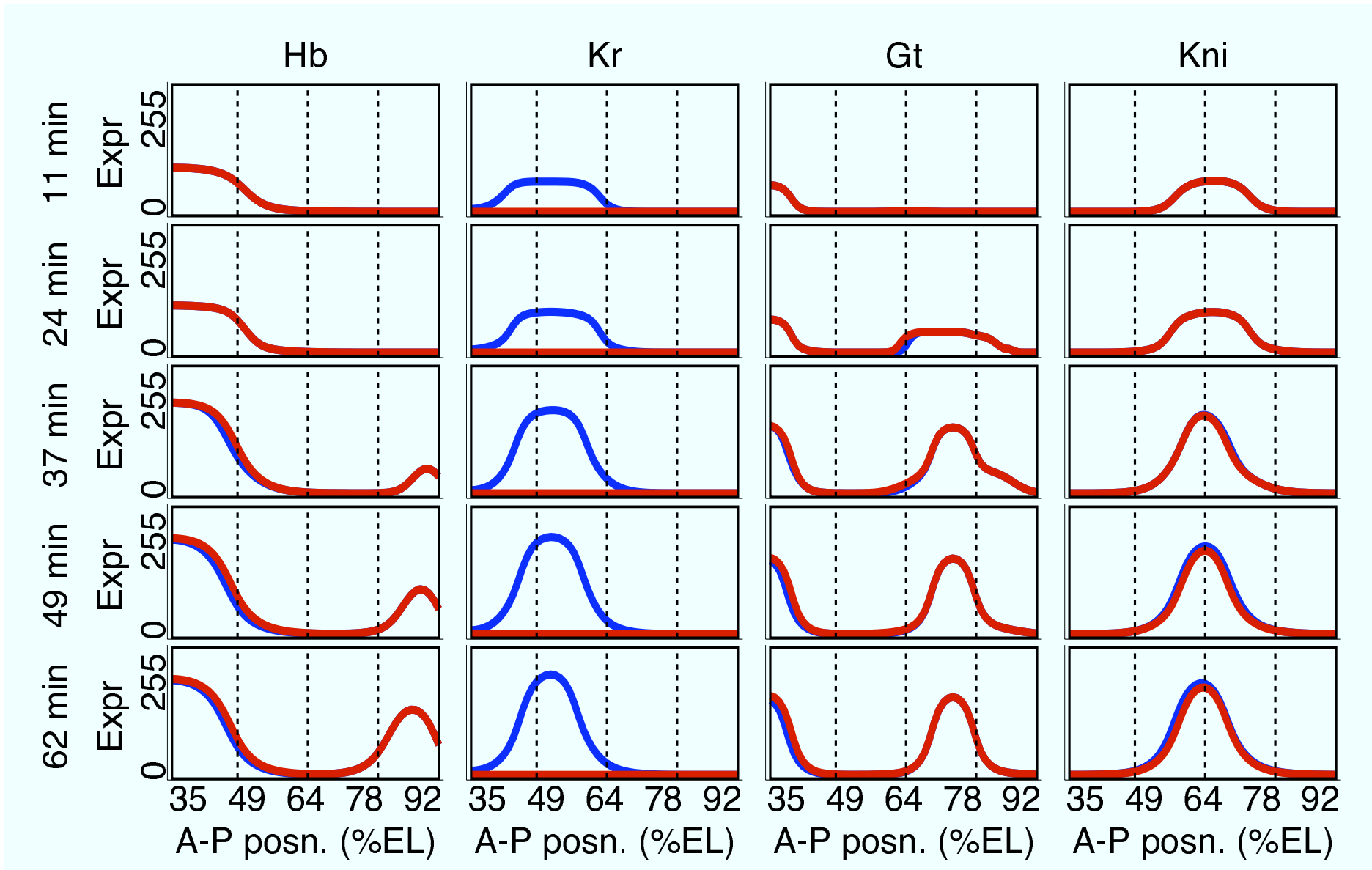

Figure 2: Simulated wild-type expression patterns from the Unc-Logic model (blue) and expression patterns predicted for a *Kr*- embryo.

References

[1] Clyde DE, Corado MSG, Wu X, Paré A, Papatsenko D, et al. (2003) A self-organizing system of repressor gradients establishes segmental complexity in *Drosophila*. Nature 426:849–853.

[2] Jäckle H, Tautz D, Schuh R, Seifert E, Lehmann R (1986) Cross-regulatory interactions among the gap genes of *drosophila*. Nature 324:668–670.

[3] Kraut R, Levine M (1991) Spatial regulation of the gap gene *giant* during *Drosophila* development. Development 111:601–609.

[4] Kraut R, Levine M (1991) Mutually repressive interactions between the gap genes *giant* and *krüppel* define middle body regions of the *drosophila* embryo. Development 111:611–621.

[5] Pankratz MJ, Hoch M, Seifert E, Jäckle H (1989) *Krüppel* requirement for *knirps* enhancement reflects overlapping gap gene activities in the *Drosophila* embryo. Nature 341:337–340.

[6] Capovilla M, Eldon ED, Pirrotta V (1992) The *giant* gene of *Drosophila* encodes a b-ZIP DNA-binding protein that regulates the expression of other segmentation gap genes. Development 114:99–112.
